# Meta-analysis of the effectiveness of fecal microbiota transplantation in the treatment of metabolic-associated fatty liver disease: A systematic review based on liver inflammation indicators and fat content

**DOI:** 10.1097/MD.0000000000046886

**Published:** 2026-01-02

**Authors:** Chunyan He, Fan Zhou, Xiangming Fang

**Affiliations:** a Department of Endocrinology, Puren Hospital Affiliated to Wuhan University of Science and Technology, Wuhan, China; b Department of Medical Biomolecular, Puren Hospital Affiliated to Wuhan University of Science and Technology, Wuhan, China; c Department of Gastroenterology, Puren Hospital Affiliated to Wuhan University of Science and Technology, Wuhan, China.

**Keywords:** ALT, AST, fecal microbiota transplantation, meta-analysis, metabolic-associated fatty liver disease, proton density fat fraction

## Abstract

**Background:**

Metabolic-associated fatty liver disease (MASLD) affects over 25% of the global population, progressing from hepatic steatosis to fibrosis. Current therapies show limited efficacy, and gut microbiota dysbiosis via the gut–liver axis highlights fecal microbiota transplantation (FMT) as a novel intervention.

**Methods:**

Following preferred reporting items for systematic reviews and meta-analyses guidelines, 8 randomized controlled trials were systematically selected from PubMed, Cochrane, Embase, and Web of Science (inception to September 2025). MASLD patients receiving FMT (any protocol) versus standard care were evaluated for alanine aminotransferase (ALT), aspartate aminotransferase (AST), proton density fat fraction, and body mass index (BMI). Risk of bias was assessed using Cochrane ROB 1.0.

**Results:**

FMT significantly reduced ALT (mean difference [MD] = ‐6.81, 95% confidence interval [‐10.29, −3.33], *P* = .0001) and AST (MD = ‐7.13, [‐10.45, −3.80], *P* < .0001) versus standard care. Subgroup analysis revealed greater ALT improvement in patients aged <50 years (MD = ‐14.00, [‐22.79, −5.20], *P* = .002). Proton density fat fraction decreased markedly (MD = ‐3.50, [‐5.12, −1.87], *P* < .0001), while BMI showed no significant change (MD = ‐0.69, [‐1.49, 0.11], *P* = .09).

**Conclusion:**

FMT effectively improves hepatic inflammation and steatosis in MASLD, with age modulating ALT response. Lack of BMI improvement suggests localized liver effects rather than systemic metabolic impact, supporting FMT as a targeted adjunctive therapy.

## 1. Introduction

Metabolic fatty liver disease (MASLD), as one of the most common chronic liver diseases in the world, its prevalence rate is rising sharply with the prevalence of obesity, type 2 diabetes and metabolic syndrome.^[1–3]^ The global prevalence rate has exceeded 25%, constituting an increasingly severe public health burden.^[4]^ The disease spectrum of MASLD covers from simple hepatic steatosis to metabolic dysfunction-associated steatohepatitis (MASH), which can further progress to liver fibrosis, cirrhosis, and even hepatocellular carcinoma.^[5,6]^ The continuous increase in liver inflammation and fat content is not only a core feature of the pathological process of MASLD, but also a key indicator for assessing disease severity, predicting disease progression, and evaluating treatment efficacy.^[7]^ At present, lifestyle intervention is the first-line strategy for MASLD management, but its long-term compliance is poor and its effectiveness is limited.^[8]^ Although drugs targeting metabolic complications have shown potential to improve liver steatosis and inflammation, no drug has been officially approved for the treatment of MASH itself, and the safety, accessibility, and cost-effectiveness of long-term use still need to be considered.^[9]^ Therefore, exploring safe and effective new treatment strategies to improve liver inflammation and steatosis is crucial for delaying the progression of MASLD, improving patient prognosis and quality of life.

In recent years, the role of gut microbiota imbalance in the occurrence and development of MASLD has been increasingly recognized.^[10]^ Numerous studies have shown that patients with MASLD exhibit significant changes in the composition and function of their gut microbiota, characterized by reduced microbiota diversity, decreased short chain fatty acid producing bacteria, increased pro-inflammatory bacteria, and impaired intestinal barrier function.^[11–13]^ This dysregulated state can mediate various pathophysiological processes through the “gut liver axis,” such as increasing endotoxin translocation into the liver, activating liver natural immune receptors, inducing chronic low-grade inflammation, promoting liver fat synthesis and insulin resistance, ultimately driving liver fat accumulation, inflammatory response, and fibrosis.^[14]^ Based on this, targeted treatment strategies for gut microbiota, especially fecal microbiota transplantation (FMT), as a therapeutic approach aimed at reshaping the balance of gut microbiota, provide new ideas for the treatment of MASLD.^[15]^

FMT refers to the transplantation of fecal microbiota from a healthy donor into the patient’s intestine to restore its normal gut microbiota composition and function.^[16]^ The significant success of FMT in the treatment of recurrent Clostridium difficile infections has sparked researchers’ interest in exploring its potential applications in other diseases associated with gut microbiota dysbiosis.^[17]^ Preliminary animal experiments and clinical studies suggest that FMT may improve MASLD through multiple mechanisms: restoring gut microbiota diversity, enhancing gut barrier function, reducing endotoxemia, regulating bile acid metabolism, improving insulin sensitivity, and alleviating liver inflammation and steatosis.^[18–20]^ However, current clinical studies on FMT treatment for MASLD are mostly exploratory trials or preliminary reports with small sample sizes, and their results exhibit certain heterogeneity. In terms of evaluating therapeutic efficacy, liver inflammation markers and liver fat content are the most commonly used noninvasive indicators to reflect the degree of liver injury and steatosis, while body mass index (BMI), as an important metabolic parameter, is also frequently monitored for its changes.^[21,22]^ Although some studies have reported that FMT has a positive effect on improving these indicators, the overall strength, consistency, and magnitude of the evidence are still unclear.

Given the dispersion and limitations of existing research, there is an urgent need to integrate existing evidence through systematic reviews and meta-analyses to provide a more comprehensive and objective evaluation of the effectiveness of FMT in treating MASLD. The aim of this study is to conduct a quantitative and combined analysis of the effects of FMT on liver inflammation, liver fat content, and BMI in MASLD patients through systematic retrieval and rigorous screening of relevant literature. Through this study, it is expected to provide more reliable scientific basis for the clinical application of FMT in the field of MASLD treatment, and provide reference for further research directions.

## 2. Materials and methods

This meta-analysis followed the preferred reporting items for systematic reviews and meta-analyses.^[23]^ This study is a review article and the ethical statement is not applicable.This study was approved by the Ethics Committee of Puren Hospital Affiliated to Wuhan University of Science and Technology.

### 2.1. Data sources and retrieval

An inclusive literature was searched in PubMed, Cochrane, Embase and web of science databases, respectively, from the date of establishment to September 3, 2025. Taking PubMed database as an example, the specific retrieval strategy is: ((metabolic-associated fatty liver disease) OR (MAFLD) OR (nonalcoholic fatty liver disease) OR (non-alcoholic fatty liver disease) OR (fatty liver, nonalcoholic) OR (fatty livers, nonalcoholic) OR (liver, nonalcoholic fatty) OR (livers, nonalcoholic fatty) OR (nonalcoholic fatty liver) OR (nonalcoholic fatty livers) OR (NAFLD) OR (nonalcoholic fatty liver disease) OR (nonalcoholic steatohepatitis) OR (nonalcoholic steatohepatitides) OR (steatohepatitides, nonalcoholic) OR (steatohepatitis, nonalcoholic)) AND ((fecal microbiota transplantation) OR (fecal microbiota transplantations) OR (microbiota transplantation, fecal) OR (microbiota transplantations, fecal) OR (transplantation, fecal microbiota) OR (transplantations, fecal microbiota) OR (fecal microbiota transplant) OR (fecal microbiota transplants) OR (microbiota transplant, fecal) OR (microbiota transplants, fecal) OR (transplant, fecal microbiota) OR (transplants, fecal microbiota) OR (fecal microbiome transplantation) OR (fecal microbiome transplantations) OR (microbiome transplantation, fecal) OR (microbiome transplantations, fecal) OR (transplantation, fecal microbiome) OR (transplantations, fecal microbiome) OR (fecal transplant) OR (fecal transplants) OR (transplant, fecal) OR (transplants, fecal) OR (donor feces infusion) OR (donor feces infusions) OR (feces infusion, donor) OR (feces infusions, donor) OR (infusion, donor feces) OR (infusions, donor feces) OR (fecal transplantation) OR (fecal transplantations) OR (transplantation, fecal) OR (transplantations, fecal) OR (intestinal microbiota transfer) OR (intestinal microbiota transfers) OR (microbiota transfer, intestinal) OR (microbiota transfers, intestinal) OR (transfer, intestinal microbiota) OR (transfers, intestinal microbiota) OR (intestinal microbiota transplantation) OR (intestinal microbiota transplantations) OR (microbiota transplantation, intestinal) OR (microbiota transplantations, intestinal) OR (transplantation, intestinal microbiota) OR (transplantations, intestinal microbiota) OR (intestinal microbiome transplantation) OR (intestinal microbiome transplantations) OR (microbiome transplantation, intestinal) OR (microbiome transplantations, intestinal) OR (transplantation, intestinal microbiome) OR (transplantations, intestinal microbiome) OR (intestinal microbiota transplant) OR (intestinal microbiota transplants) OR (microbiota transplant, intestinal) OR (microbiota transplants, intestinal) OR (transplant, intestinal microbiota) OR (transplants, intestinal microbiota) OR (intestinal microbiome transfer) OR (intestinal microbiome transfers) OR (microbiome transfer, intestinal) OR (microbiome transfers, intestinal) OR (transfer, intestinal microbiome) OR (transfers, intestinal microbiome) OR (fecal microbiota transfer) OR (fecal microbiota transfers) OR (microbiota transfer, fecal) OR (microbiota transfers, fecal) OR (transfer, fecal microbiota) OR (transfers, fecal microbiota) OR (intestinal microbiome transplant) OR (intestinal microbiome transplants) OR (microbiome transplant, intestinal) OR (microbiome transplants, intestinal) OR (transplant, intestinal microbiome) OR (transplants, intestinal microbiome)). The effect sizes of each study and the population were calculated under a fixed effects model with a 95% confidence interval (CI).

### 2.2. Inclusion and exclusion criteria of the literature

*Inclusion criteria*: The research type is randomized controlled trial (RCT); The research subjects are MASLD patients who meet the above diagnostic criteria; The intervention measure is FMT (unlimited route and frequency) compared to standard treatment; The study needs to report baseline and post intervention data (mean ± standard deviation or convertible data) for at least 2 predetermined outcome measures (alanine aminotransferase [ALT], aspartate aminotransferase [AST], proton density fat fraction [PDFF], BMI); The language is limited to English; The full text is available.

*Exclusion criteria*: Patients with other chronic liver diseases (such as viral hepatitis, autoimmune liver disease, and decompensated cirrhosis) or severe systemic diseases (end-stage renal disease, malignant tumors); The study involved FMT combined with other microbial interventions (such as antibiotic pretreatment, specific probiotics) but did not establish a separate FMT subgroup; The research type is review, case report, conference abstract, or animal experiment; Incomplete data; Repeatedly published literature; Non Chinese English literature.

### 2.3. Quality assessment and data extraction

Two researchers independently screened literature, extracted data, and cross checked. In case of disagreement, it shall be resolved through consultation with the third researcher. When screening literature, first read the title and abstract, and after excluding obviously unrelated literature, further read the entire text to determine whether it is ultimately included. The content of data extraction includes: Basic information included in the study, including author, publication date, country or region, etc; The basic characteristics of the research object include patient age, gender, sample size, etc; Specific details of intervention measures, including the dosage of CDK4/6 inhibitors; Key elements of bias risk assessment; The outcome measures and outcome measurement data of concern. Conduct bias risk assessment using the Cochrane Handbook’s Risk of Bias Assessment Tool (ROB 1.0) for RCTs. The main criteria include: generation of random sequences; Allocation concealment; Blinding researchers and participants; Turn a blind eye to the evaluators of the results; Data integrity; Selective reporting bias; And other biases. Answer with “low risk,” “high risk,” and “unclear.”

### 2.4. Statistical analysis

Perform meta-analysis using Review Manager 5.3 software (The Cochrane Collaboration, London, UK). The time event variable uses hazard ratio as the effect analysis statistic, and the binary variable uses relative risk as the effect analysis statistic. Each effect variable provides a 95% CI.^[24]^ The heterogeneity among the included research results was analyzed using *Q* test, and the size of heterogeneity was determined by combining *I*^2^ values. If there is no statistical heterogeneity between the research results (*P* ≥ .1 and *I*^2^ ≤ 50%), a fixed effects model will be used for meta-analysis; On the contrary, further analysis of heterogeneity sources is conducted, and after excluding the influence of significant clinical heterogeneity, a random effects model is used for meta-analysis. Process studies with clinical heterogeneity using methods such as subgroup analysis or sensitivity analysis. The difference is statistically significant with *P* < .05.

## 3. Results

### 3.1. Literature search results

**Figure 1.**
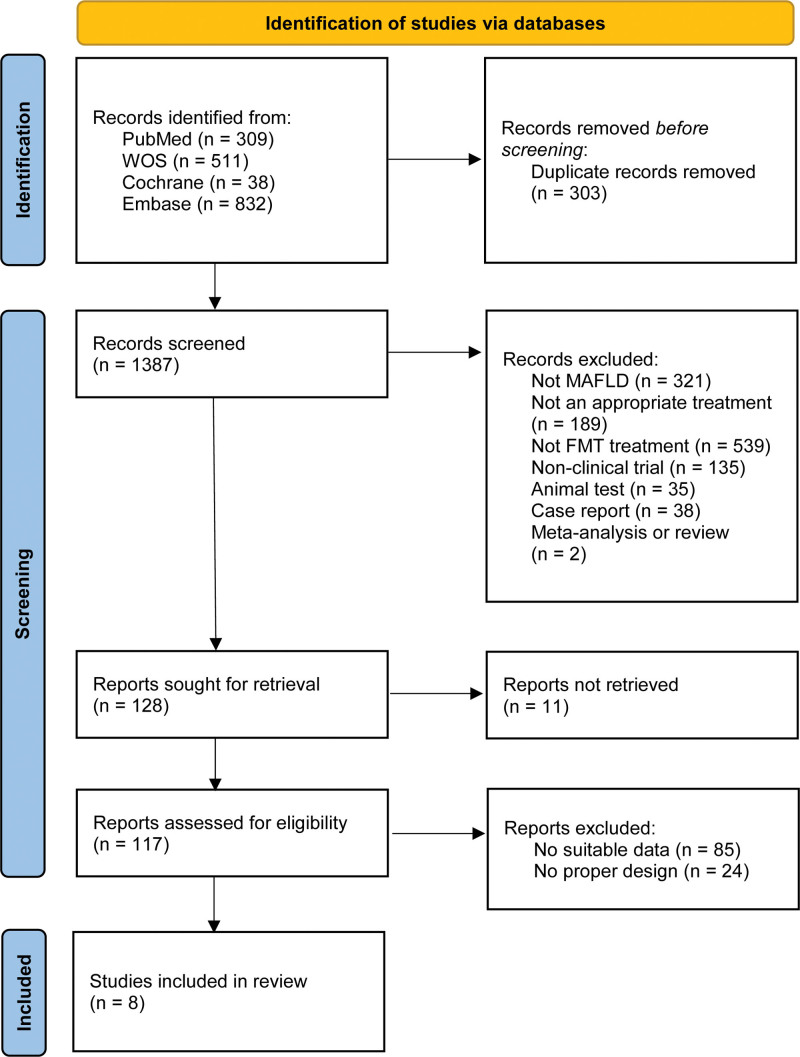
Flow chart of literature screening.

A total of 1690 relevant records were identified through the initial search. After removing duplicates and performing stepwise screening based on titles, abstracts, and full texts, 8 studies^[25–32]^ were finally included in the meta-analysis. The detailed screening process is illustrated in Figure 1.

### 3.2. Basic characteristics and quality assessment of the included literature

**Figure 2.**
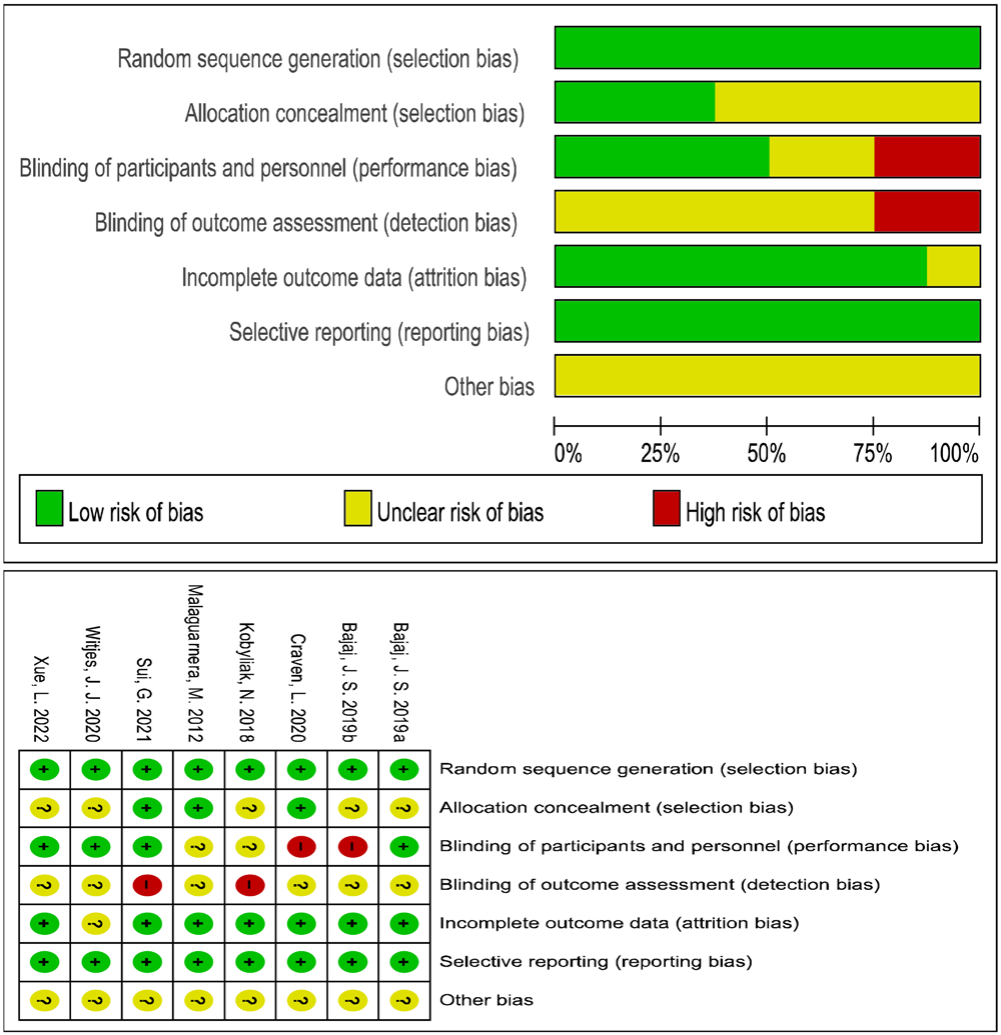
Results of bias risk assessment of the included studies.

A total of 8 English papers were included, and all patients met the internationally recognized diagnostic criteria for MASLD, which were diagnosed through liver tissue biopsy (histological confirmation of hepatic steatosis ≥5%) or noninvasive imaging examination (such as MRI-PDFF ≥ 5%), and excessive alcohol consumption and other clear causes of hepatic steatosis were excluded. The experimental group received FMT treatment strategy, while the control group received standard of care (SOC) strategy. The patient’s baseline features should include complete data on ALT, AST, PDFF, or BMI. Use Cochrane 5.1.0 scale to evaluate the quality of literature. The basic characteristics and quality assessment of the included literature (Table 1). The bias risk assessment is shown in Figure 2.

**Table 1:** Basic characteristics and quality assessment of the included studies (n = 8).

Include studies	Country/region	Research subject	Number		Age		Intervention	Control
			T	C	T	C		
Bajaj et al^[25]^	America	MAFLD	7	6	–	–	FMT	SOC
Bajaj et al^[26]^	America	MAFLD	10	10	63.3	62.2	FMT	SOC
Craven et al^[27]^	Canada	MAFLD	6	15	47.6	47.5	FMT	SOC
Kobyliak et al^[28]^	Ukraine	MAFLD	30	28	53.4	57.3	FMT	SOC
Malaguarnera et al^[29]^	Italy	MAFLD	34	32	46.9	46.7	FMT	SOC
Sui et al^[30]^	China	MAFLD	59	32	–	–	FMT	SOC
Witjes et al^[31]^	Netherlands	MAFLD	11	10	48.5	51.2	FMT	SOC
Xue et al^[32]^	China	MAFLD	47	28	57.3	60.2	FMT	SOC

T: treatment group, C: control group.

FMT = fecal microbiota transplantation, MASLD = metabolic-associated fatty liver disease, SOC = standard of care.

### 3.3. Meta-analysis results

#### 3.3.1. Alanine aminotransferase

**Figure 3.**
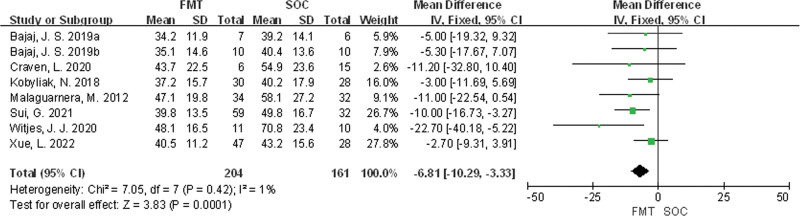
Meta-analysis of ALT comparison between FMT group and SOC group. ALT = alanine aminotransferase, FMT = fecal microbiota transplantation, SOC = standard of care.

Eight studies^[25–32]^ were included in total. There was no statistical heterogeneity among the studies (*P* = .42, *I*^2^ = 1%), and a fixed effects model was used for meta-analysis. The results showed that there was a statistically significant difference in ALT levels between the FMT group and the SOC group (mean difference [MD] = −6.81, 95% CI [−10.29, −3.33], *P* = .0001) in Figure 3.

**Figure 4.**
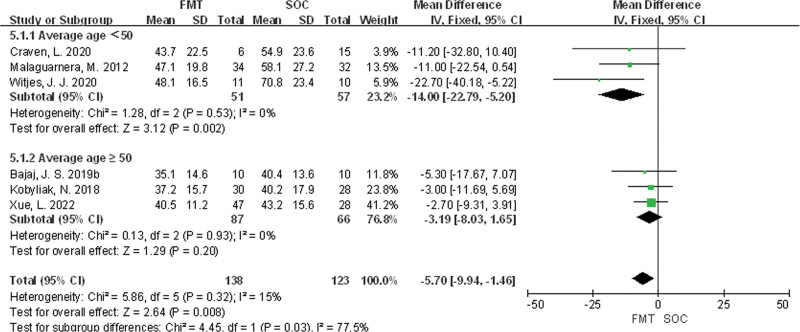
Comparison of the effects of different age on ALT in MASLD patients. ALT = alanine aminotransferase, MASLD = metabolic-associated fatty liver disease.

Subgroup analysis was conducted based on the average age of patients, and the results showed that there was a statistically significant difference in ALT levels between the FMT and SOC groups in studies with an average age <50 (MD = −14.00, 95% CI [−22.79, −5.20], *P* = .002), while there was no statistically significant difference between the FMT and SOC groups in studies with an average age ≥50 (MD = −3.19, 95% CI [−8.03, 1.65], *P* = .20), as shown in Figure 4.

#### 3.3.2. Aspartate aminotransferase

**Figure 5.**
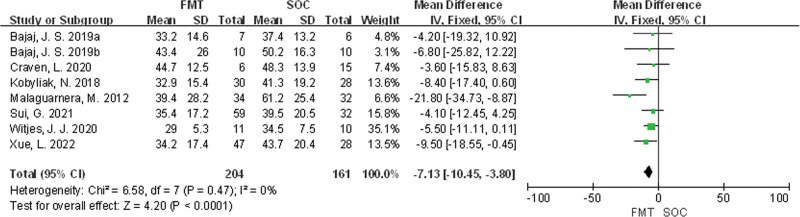
Meta-analysis of AST comparison between FMT group and SOC group. AST = aspartate aminotransferase, FMT = fecal microbiota transplantation, SOC = standard of care.

Eight studies^[25–32]^ were included in total. There was no statistical heterogeneity among the studies (*P* = .47, *I*^2^ = 0%), and a fixed effects model was used for meta-analysis. The results showed that there was a statistically significant difference in AST levels between the FMT group and the SOC group (MD = −7.13, 95% CI [−10.45, −3.80], *P* < .0001) in Figure 5.

**Figure 6.**
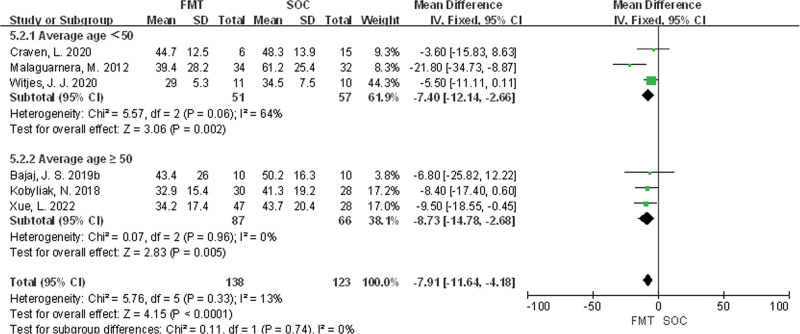
Comparison of the effects of different age on AST in MASLD patients. AST = aspartate aminotransferase, MASLD = metabolic-associated fatty liver disease.

Subgroup analysis was conducted based on the average age of patients, and the results showed that there was a statistically significant difference in AST levels between the FMT and SOC groups in studies with an average age <50 (MD = −7.40, 95% CI [−12.14, −2.66], *P* = .002), while there was also a statistically significant difference in AST levels between the FMT and SOC groups in studies with an average age ≥50 (MD = −8.73, 95% CI [−14.78, 2.68], *P* = .005), as shown in Figure 6.

#### 3.3.3. Proton density fat fraction

**Figure 7.**
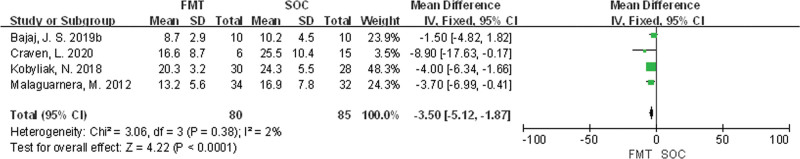
Meta-analysis of PDFF comparison between FMT group and SOC group. FMT = fecal microbiota transplantation, PDFF = proton density fat fraction, SOC = standard of care.

Four studies^[26–29]^ were included in total. There was no statistical heterogeneity among the studies (*P* = .38, *I*^2^ = 2%), and a fixed effects model was used for meta-analysis. The results showed that there was a statistically significant difference in PDFF levels between the FMT group and the SOC group (MD = −3.50, 95% CI [−5.12, −1.87], *P* < .0001) in Figure 7.

#### 3.3.4. Body mass index

**Figure 8.**
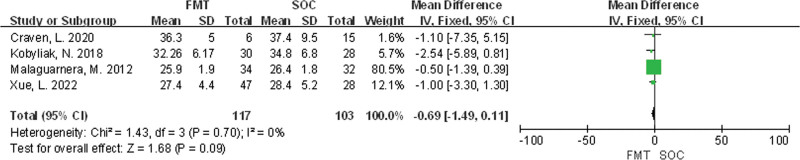
Meta-analysis of BMI comparison between FMT group and SOC group. FMT = fecal microbiota transplantation, SOC = standard of care.

Four studies^[27–29]^ were included in total. There was no statistical heterogeneity among the studies (*P* = .70, *I*^2^ = 0%), and a fixed effects model was used for meta-analysis. The results showed that there was no statistically significant difference in BMI levels between the FMT group and the SOC group (MD = −0.69, 95% CI [−1.49, 0.11], *P* = .09) in Figure 8.

### 3.4. Publication bias

**Figure 9.**
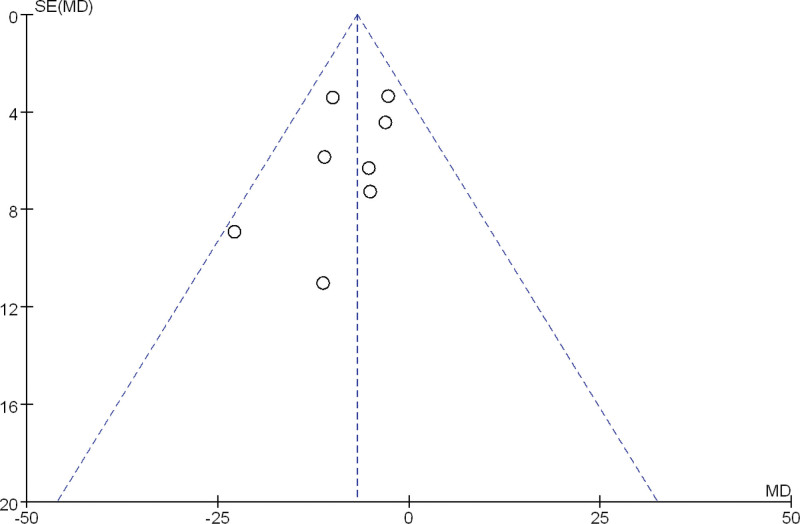
Funnel plot of publication bias in ALT between FMT group and SOC group. ALT = alanine aminotransferase, FMT = fecal microbiota transplantation, SOC = standard of care.

**Figure 10.**
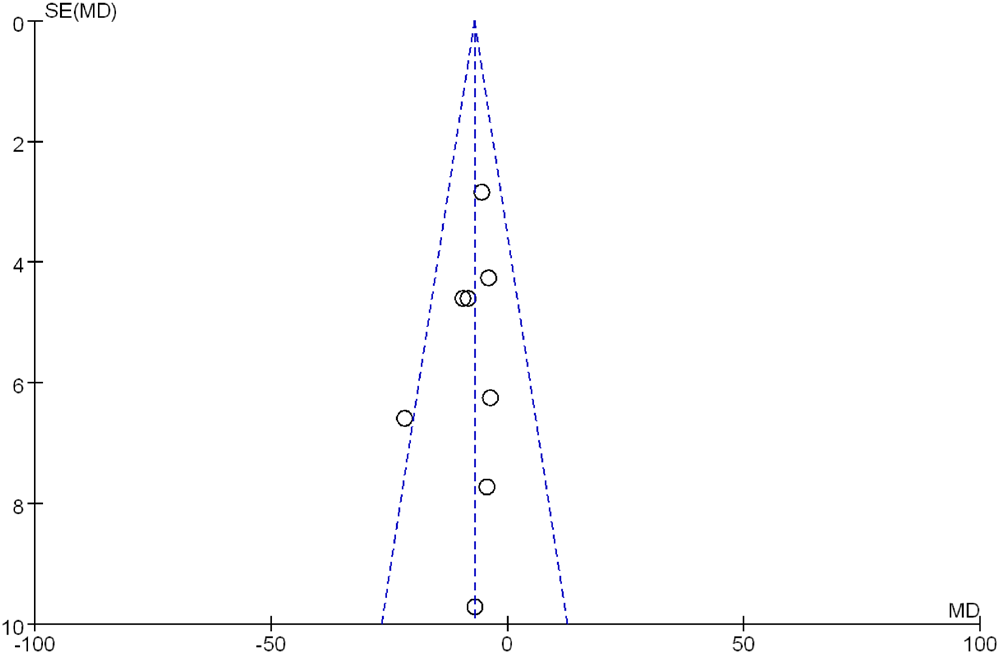
Funnel plot of publication bias in AST between FMT group and SOC group. AST = aspartate aminotransferase, FMT = fecal microbiota transplantation, SOC = standard of care.

A funnel plot was drawn for the 2 outcome measures ALT and AST for publication bias testing. The results showed that most of the scatter points were concentrated in the range of MD −50 to 50, and the distribution of each study point was basically symmetrical, indicating a low possibility of publication bias. See Figures 9 and 10.

## 4. Discussion

This study comprehensively evaluated for the first time the effects of FMT on liver inflammation indicators (ALT, AST), liver fat content indicators (PDFF), and metabolic indicators (BMI) in MASLD patients through systematic review and meta-analysis. The results showed that compared with SOC, FMT could significantly reduce the levels of ALT, AST, and PDFF in MASLD patients, but the improvement in BMI did not reach statistical significance. This discovery provides important evidence to support the improvement of MASLD liver pathological process by FMT.

In the indicator system for evaluating the efficacy of FMT, ALT, and AST are classic biomarkers of hepatocellular injury, primarily reflecting the integrity of hepatocyte membranes and the degree of inflammatory activity.^[33]^ This study found that FMT significantly improved both indicators, suggesting that FMT may alleviate hepatic inflammation by modulating the gut–liver axis.^[34,35]^ Notably, subgroup analysis revealed that age was an important modifier of ALT response: patients with an average age of <50 years exhibited a more pronounced reduction in ALT levels. This may be biologically explained by several factors. Younger individuals generally have higher gut microbiota plasticity, allowing for more efficient colonization and functional integration of donor microbes. They also tend to have a shorter duration and lower cumulative burden of metabolic dysfunction, which may make hepatic inflammatory pathways more reversible. In addition, age-related immune senescence and alterations in bile acid signaling in older adults could attenuate the microbiota–liver crosstalk and thus limit the anti-inflammatory effect of FMT. In contrast, AST improvement was significant across all age groups, suggesting that the mitochondrial repair effects of FMT may be independent of age-related host factors. As an imaging-based marker that directly quantifies hepatic fat content, the significant reduction in PDFF further confirms that FMT can effectively mitigate hepatic steatosis, consistent with experimental findings that FMT reduces hepatic lipid deposition and restores lipid metabolism homeostasis in animal models.^[19]^ By comparison, BMI—a systemic metabolic indicator—showed no significant change, supporting the notion that FMT acts primarily on the local hepatic microenvironment rather than inducing broad systemic metabolic remodeling.^[36]^

The joint analysis of 4 outcome indicators revealed their intrinsic connections and complementary values: ALT/AST, as a dynamic inflammatory indicator, can sensitively reflect the short-term anti-inflammatory effect of FMT; PDFF confirms substantial improvement in fat degeneration from a structural perspective; The negative result of BMI suggests that simple FMT intervention may not be sufficient to reverse obesity related systemic metabolic disorders.^[37,38]^ This is consistent with the multifactorial pathogenesis of MASLD—although gut microbiota dysbiosis plays a central role in intrahepatic inflammation and steatosis, weight control still requires comprehensive management such as lifestyle interventions.^[39]^ Therefore, future research should explore the synergistic effects of FMT combined with diet/exercise therapy. From a clinical translation perspective, ALT/AST, as a sensitive biomarker, can quickly reflect the anti-inflammatory effect of FMT and provide immediate basis for dynamically adjusting treatment plans; PDFF objectively confirms the reversal effect of FMT on the core pathological changes of MASLD by quantifying the degree of hepatic steatosis, and its noninvasive nature makes it an ideal alternative to liver biopsy; The result of no significant improvement in BMI highlights the characteristic of FMT that focuses more on local liver microenvironment regulation rather than systemic metabolic regulation. This targeted difference suggests that FMT may have unique therapeutic value for lean MASLD patients with normal BMI; For obese patients, combined lifestyle interventions are needed to achieve comprehensive metabolic management.

The limitations of this study are reflected in several aspects. First, the selection of outcome measures has inherent constraints: Although ALT and AST are widely used, they lack specificity and cannot distinguish the source of inflammation; PDFF quantifies fat content but does not evaluate inflammatory activity or fibrosis, which are key determinants of MASLD prognosis; BMI, as an indicator of obesity, fails to distinguish fat distribution, which may obscure potential FMT effects on body composition; and Histological endpoints were not included, making it difficult to directly evaluate improvements in MASH. Furthermore, there was considerable heterogeneity in FMT regimens among the included studies, including differences in delivery routes (oral capsules, colonoscopy, or nasoduodenal infusion), dosing frequency, and donor selection, which may have influenced the pooled outcomes. Most studies also had relatively short follow-up durations, preventing assessment of the long-term efficacy and safety of FMT in MASLD. In addition, the assessment of publication bias was limited due to the small number of included trials. Finally, the sample sizes in several studies were modest, which could potentially overestimate the treatment effect. Future research should therefore focus on designing large-scale, standardized, and long-term RCTs to confirm these findings.

Despite the aforementioned limitations, this study confirms the clinical potential of FMT in improving liver inflammation and steatosis in MASLD. Especially for young patients, FMT may serve as an adjuvant therapy to delay disease progression. Future research should focus on the following directions: Developing a standardized formula for FMT microbiota targeting MASLD; Combining multiple omics techniques to elucidate the mechanism of action of FMT; Exploring the synergistic effects of FMT in combination with metabolic drugs; Extend follow-up time to evaluate the long-term efficacy and safety of FMT.

## Author contributions

**Conceptualization:** Chunyan He, Fan Zhou, Xiangming Fang.

**Data curation:** Chunyan He, Fan Zhou, Xiangming Fang.

**Formal analysis:** Chunyan He, Fan Zhou, Xiangming Fang.

**Funding acquisition:** Chunyan He, Fan Zhou, Xiangming Fang.

**Investigation:** Chunyan He, Fan Zhou, Xiangming Fang.

**Writing – original draft:** Chunyan He, Fan Zhou, Xiangming Fang.

**Writing – review & editing:** Chunyan He, Fan Zhou, Xiangming Fang.
